# Landscape of TP53 Alterations in Chronic Lymphocytic Leukemia *via* Data Mining Mutation Databases

**DOI:** 10.3389/fonc.2022.808886

**Published:** 2022-02-16

**Authors:** Thierry Soussi, Panagiotis Baliakas

**Affiliations:** ^1^ Department of Immunology, Genetics and Pathology, Uppsala University, Uppsala, Sweden; ^2^ Sorbonne Université, UPMC Univ Paris 06, Paris, France

**Keywords:** mutation database, TP53 mutation, chronic lymphocytic leukemia, variant classification guidelines, genetic analysis model

## Abstract

Locus-specific databases are invaluable tools for both basic and clinical research. The extensive information they contain is gathered from the literature and manually curated by experts. Cancer genome sequencing projects generate an immense amount of data, which are stored directly in large repositories (cancer genome databases). The presence of a *TP53* defect (17p deletion and/or *TP53* mutations) is an independent prognostic factor in chronic lymphocytic leukemia (CLL) and *TP53* status analysis has been adopted in routine clinical practice. For that reason, *TP53* mutation databases have become essential for the validation of the plethora of *TP53* variants detected in tumor samples. *TP53* profiles in CLL are characterized by a great number of subclonal *TP53* mutations with low variant allelic frequencies and the presence of multiple minor subclones harboring different *TP53* mutations. In this review, we describe the various characteristics of the multiple levels of heterogeneity of *TP53* variants in CLL through the analysis of *TP53* mutation databases and the utility of their diagnosis in the clinic.

## Introduction

**Figure 1:**
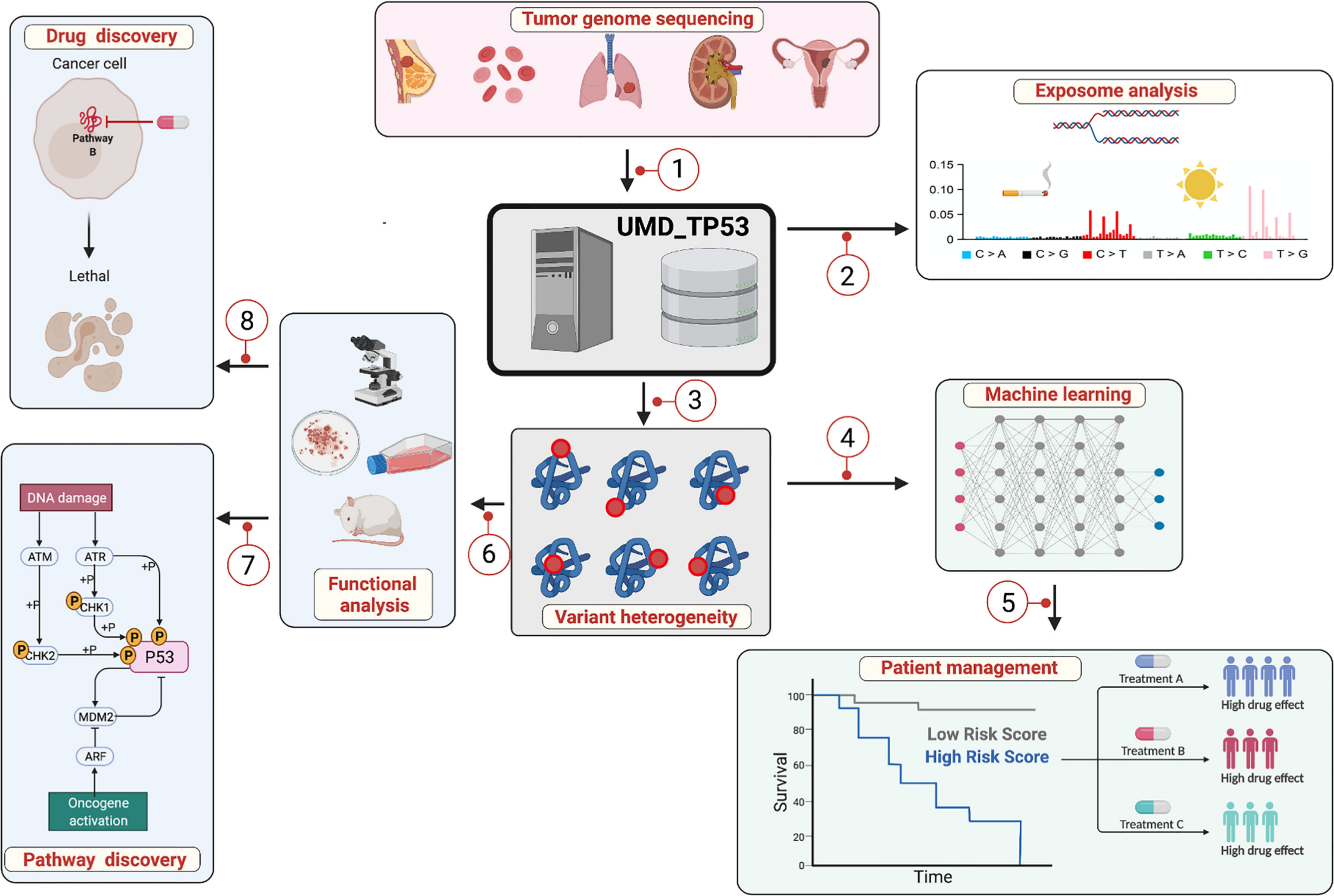
The locus-specific database UMD_*TP53*: a central hub for multifactorial analysis. 1: *TP53* variants and patient information are collected and stored in a relational database specifically developed for the storage and the analysis of genetic variants. 2: Exposome analysis: influence of the external and internal environment on the landscape of mutational events to identify the links between exposure to various types of carcinogens, specific mutational events in the *TP53* gene and the development of specific cancers. 3: More than 7,000 different *TP53* variants have been discovered in various types of cancer with heterogeneous LOF and GOF. 4: Multiple bioinformatics tools, including machine learning, have been developed to predict and classify *TP53* variants. 5: Genome-based prognostic biomarkers can be used for several cancer types for potential incorporation into clinical prognostic staging systems or practice guidelines such as *TP53* and CLL. 6: Analysis of *TP53* variants points to the various functional domains of the protein essential for tumor suppression. 7: Functional analysis has led to the identification of the multiple pathways regulated by *TP53*. 8: Small molecules have been developed that specifically target missense *TP53* variants and restore p53 transcriptional activity, thereby enabling tumor regression. Although this figure describes the *TP53* database, the various aspects can be applied to other genes as well.

In 1956, Ingram used protein sequencing to provide the first demonstration of a severe disease (human sickle-cell anemia in that work) resulting from a single amino acid substitution (1). Since then, it has been largely demonstrated that gene mutations are the basis for most genetic diseases. The development of DNA sequencing and molecular cloning technologies in the late 1970s contributed greatly to the identification of genes involved in both monogenic and polygenic disorders, including complex diseases like cancer (2). The alterations occurring in those genes are numerous and variable in nature, ranging from point mutations to large deletions or translocations. Moreover, the task of reporting, storing, classifying and analyzing them has been a major challenge (3). To provide a pertinent response to this latter, locus-specific databases (LSDBs) have been developed (**Figure 1**). Although intended for single genes, LSDBs do offer great accuracy as they are curated manually by experts in the field (4, 5). They also provide information that can be used for large-scale analyses and often include structural, functional or evolutionary data (6). For constitutional mutations associated with a genetic syndrome, several LSDBs also include phenotypic data useful for the study of genotype-phenotype correlation (7).

Genomic studies of tumor samples in the pre-genomic era were focused either on a small number of genes analyzed in large patient cohorts, or on a more significant number of genes but in only a few tumors. Indeed, large-scale analyses combining a multitude of genes and tumors represented a Herculean and costly task. The development of high-throughput methodologies capable of sequencing an entire genome in only a few days (next generation sequencing, NGS) has radically changed the entire field of cancer biology. In the present post-genomic era, whole genome sequencing in a multitude of tumors can be performed in a matter of days. The International Cancer Genome Consortium (ICGC, http://dcc.icgc.org/), the Cancer Genome Atlas Project (TCGA, http://cancergenome.nih.gov/) and the Sanger Institute (http://www.sanger.ac.uk/) have undertaken large-scale cancer genome analyses in different types and subtypes of cancer. That work has led to the creation of large data repositories (cancer genome databases, CGDs) freely available to the entire scientific community (8–10). Both LSDBs and CGDs can be considered as central hubs linking clinical and basic research (**Figure 1**). They all make important contributions to our knowledge of the intricate pathways regulating cell fate, and our ability to identify new clinical biomarkers and develop novel therapeutic molecules.

*TP53* mutation databases are the perfect example of the successful use of these compilations of cancer associated alterations. Indeed, the *TP53* suppressor gene is the most frequently mutated gene in human cancer and analyses of these alterations have fueled basic and clinical research, leading in turn to a number of novel therapeutics currently in phase III trials (11).

## *TP53* Databases and Repositories

Although multiple *TP53* LSDBs have been created, only two, UMD_*TP53* (Universal Mutation_Database, developed by the present team) and IARC, count 30 years of *TP53* mutation analyses in various types of cancer (**Table 1**) (12, 13). Both have been regularly updated with both *TP53* variants and new tools to classify them. The IARC database was updated for the last time in 2019 and is currently awaiting transfer to a new host. The next update to the UMD_*TP53* will be performed in March 2022. It will bring a new innovative system to classify *TP53* variant pathogenicity and a new version of Seshat to analyze variants (14).

**Table 1:** TP53 mutation databases.

	UMD^1^	IARC^2^	LOVD^3^	COSMIC^4^	TCGA^5^	ICGC^6^	MSKSCC^7^	GENIE^8^
	LSDB	LSDB	LSDB	CGD	CGD	CGD	CGD	CGD
**Version**	2021R1	R20, July 2019	*TP53*:210617	v94	NA	v28	V10	V10
**Creation date**	1991	1991	2013	2004	2008	2013	2016	2016
**Last update**	2021	2019	Jun-21	May-21	Jun-21	Mar-21	Jun-21	Jun-21
**Number of entries**	170,428	29,891	676^9^	47,788	4,250	6557	3,249	4,813
**Unique variant**	8,046	4,526	400	5,705		1,961	1031	11,30
**Cell lines data**	Yes	Yes	No	Yes	No	No	No	No
**Curated publications**	6,704	2,273	6	4,129	32 studies	86 projects	NR	NR
**Online search**	Yes	Yes	No	Yes	Yes	Yes	No	Yes*
**Publication warning** ^**10**^	Yes	No	No	No	No	No	No	No
**Sex/Age/Ethnicity**	No	Yes	No	Partial	Yes	Yes	Yes	Yes
**Curation for duplicate publications** ^**11**^	Yes	Unknown	Unknown	Unknown	NR	NR	NR	NR
**Sample duplications**	No	No	No	Yes	NR	NR	NR	NR
**SNP curation**	Yes	Partial	No	Partial	Partial	Partial	Partial	Partial
**Availability of functional data**	Yes	Yes	No	No	No	No	No	No
**Availability of predictive data**	Yes	Yes	No	Yes	Yes	Yes	Yes	Yes
**ACMG criteria**	Yes	No	No	No	No	No	No	No
**Data accuracy**	Yes	Yes	unknown	Yes	Yes	Yes	Yes	Yes
**Germline mutation**	Yes	Yes	Yes	Yes	No	No	No	No
**Familial data**	No	Yes	No	No	No	No	No	No
**Availability for Download**	Yes	Yes	Yes	Yes	Yes	No*	Yes	Yes
**Submission for analysis**	No	No	Yes	No	No	No	No	No
**Current status**	Alive	on hold	Unknown	Alive	Alive	Alive	Alive	Alive
**CLL publications/cases**	179	31	0	412	0	0	6 CLL cases	235 CLL cases
**Number of *TP53* variants in CLL**	4,698	187	0	40	0	0	0	13

^1^ http://p53.fr/tp53-database/mutation-database.

^2^ https://p53.iarc.fr/.

^3^ https://databases.lovd.nl/shared/genes/TP53; LOVD database includes mostly non-pathogenic SNPs reported in population studies.

^4^ https://cancer.sanger.ac.uk/cosmic.

^5^Only the 32 PAN cancer studies (10,967 samples) are included here.

^6^ https://www.cbioportal.org/.

^7^ https://www.synapse.org/#!Synapse:syn7222066/wiki/405659; MSKSCC data were extracted from GENIE V10.0.

^8^All GENIE data except MSKSCC study.

^9^LOVD database includes mostly non-pathogenic SNPs reported in population studies.

^10^Manuscript known to includes spurious data are flagged.

^11^Multiple publications report genetic information for the same patient. *Only via https://genie.cbioportal.org/.

The number of CLL-related *TP53* mutations in the various databases is quite low except in UMD_*TP53* (**Table 1**). Because of the clinical importance of *TP53* mutations in CLL, a curated subset for that pathology, called UMD_CLL, has been added to the UMD_*TP53* database (**Figure 3A**). The latest version of UMD_CLL includes 4,698 mutations, corresponding to 3,419 samples, as patients with multiple *TP53* mutations are frequent in this disease. The characteristics of these variants are discussed in the following sections of this review.

As early as 2005, in collaboration with C. Ishioka’s group, UMD_*TP53* was updated with *TP53* functional data to improve the curation of the database and develop the first tools to assess *TP53* variant loss of function (LOF) (15, 16). These tools have shown tremendous value for distinguishing true oncogenic *TP53* variants from passenger or artifactual mutations. Data from two recent large-scale studies analyzing *TP53* LOF *via* multiple assays in mammalian cells have also been included in UMD_*TP53* to refine *TP53* variant classification (17–19). Version 1 of Seshat was released in 2018. Seshat is a web service for annotating *TP53* information derived from sequencing data. It allows the use of mutation annotation format (MAF) or variant call format (VCF) files. Seshat performs accurate variant annotations using the nomenclature of the Human Genome Variation Society and the stable *TP53* genomic reference provided by Locus Reference Genomic (14).

**Figure 2:**
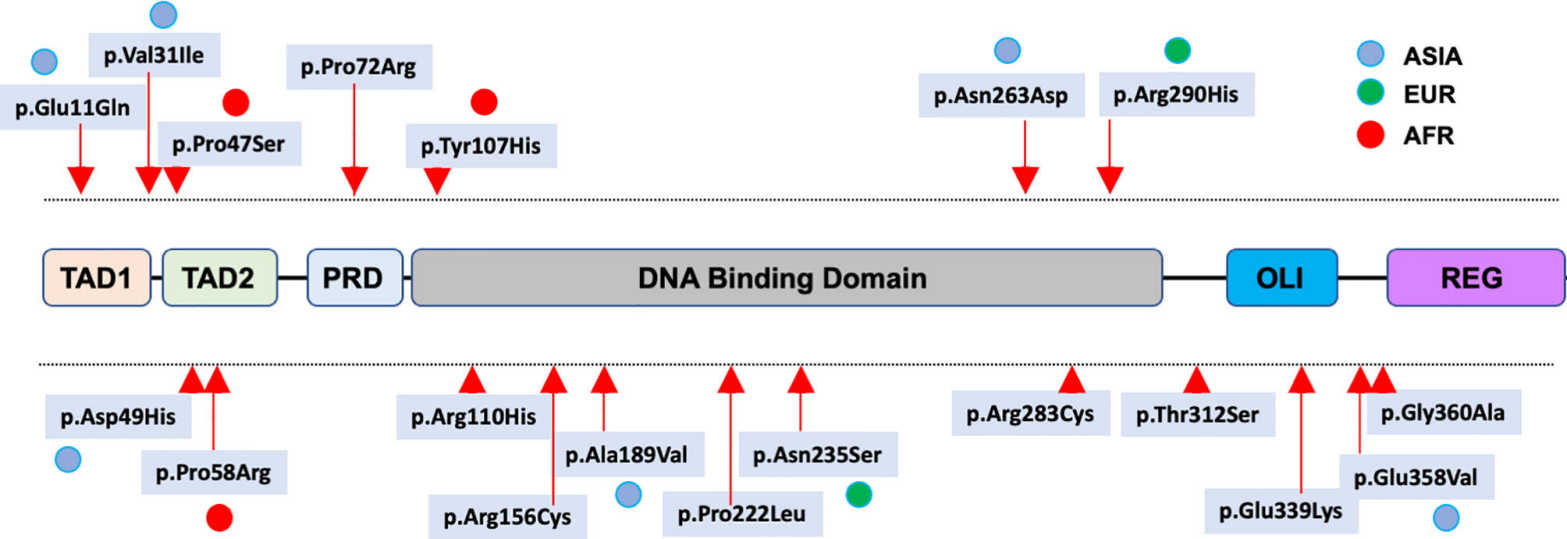
Distribution of benign missense *TP53* SNPs in the p53 protein. SNPs specific for an ethnic population are indicated by colored dots.

**Figure 3:**
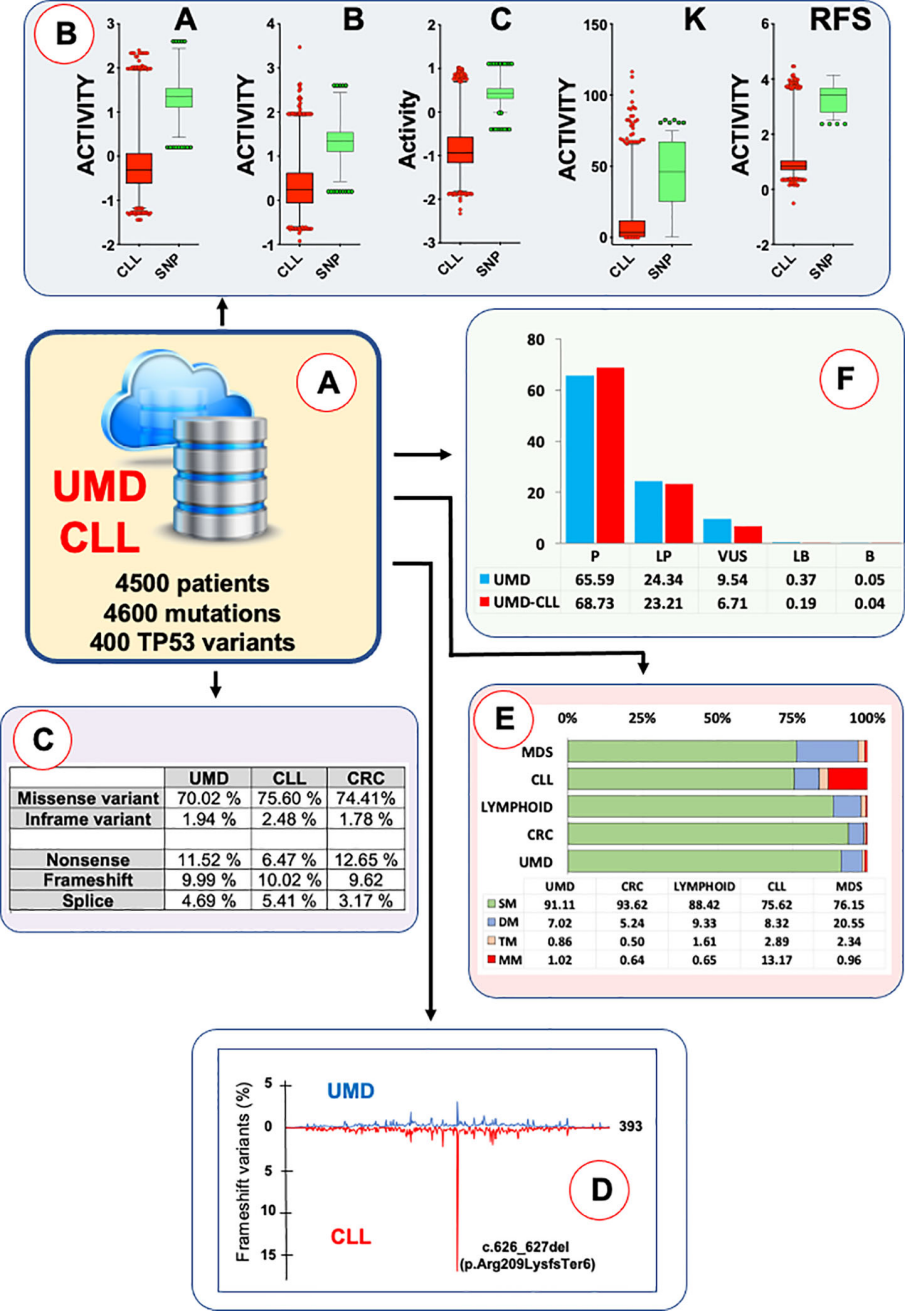
The UMD_CLL database. **(A)** *TP53* mutations from CLL patients included in UMD_*TP53* have been manually curated to correct for study duplication. For patients analyzed *via* Sanger in the nineties and *via* NGS more recently, only the more recent data were kept in the database as the sensitivity of NGS uncovered less frequent variants. **(B)** The UMD_CLL database includes three independent sets of functional data used to assess the loss of function of more than 10,000 *TP53* variants: A, B and C, data from Giacomelli et al. in mammalian cells; RFS, data from Kotler et al. in mammalian cells; K, data from Kato et al. in yeast cells. Correlation analysis and multidimensional scaling showed excellent agreement between these three sets of data (19). Each dataset has been used to compare the *TP53* variants from UMD_CLL (red) to benign *TP53* SNPs (green). **(C)** The landscape of *TP53* variants in CLL is similar to that of other types of cancer, with 78% of tumors expressing a mutant *TP53* (missense and in-frame variants) and 22% null variants (splice, nonsense and frameshift mutations); **(D)** Analysis of the distribution of *TP53* variants in TP53 protein from CLL patients showed several unusual features, such as a frameshift mutation in codon 209. See text for more details. **(E)** At least 25% of CLL patients carry at least two pathogenic *TP53* variants, and up to 13% carry more than four. This situation is shared only with myelodysplastic syndrome, where up to 20% of patients show two *TP53* variants. As half of the CLL data in UMD_*TP53* originated from Sanger analyses, it is likely that CLL intratumor heterogeneity is underestimated. **(F)** All *TP53* variants from UMD_*TP53* have been classified according to ACMG criteria. For this purpose, all newly discovered, rare, benign SNPs misidentified as pathogenic mutations have been removed from the database.

Several single nucleotide polymorphisms (SNPs) in the coding region of the *TP53* gene have been identified and extensively characterized. Among the missense SNPs, rs1042522 (p.Pro72Arg) is common in all populations across the globe. Contrastingly, rs1800371 (p.Pro47Ser) has been shown to be specific to the African population (20). Both SNPs are included in ClinVar and considered benign according to American College of Medical Genetics and Genomics (ACMG) criteria. In a recent survey, new *TP53* missense SNPs, including five variants specific to the Asian population, were identified and characterized (**Figure 2**) (21). None of these variants were found to display LOF compared to the normal *TP53* gene (**Figure 3B**) and they are now defined as bona fide benign SNPs (21). In UMD_*TP53*, these variants are specifically flagged as germline SNPs. However, other LSDBs and CGDs define several of them as somatic and potentially pathogenic variants.

The three major CGDs (ICGC, TCGA and GENIE) include data from both whole exome and whole genome sequencing of multiple tumors (**Table 1**). CGDs list fewer *TP53* variants than LSDBs do. However, the former are able to show the full pattern of mutations in a single tumor, which enables analyses that are not possible with the latter. For example, CGDs enable the identification of mutual exclusivity of genomic alterations to identify genes belonging to a same functional pathway, as they do not mutate simultaneously in a same patient (22).

## Shaping the Landscape of *TP53* Mutations in CLL

Although most DNA damage resulting from endogenous and/or exogenous insults is successfully managed by the various DNA repair mechanisms, some does escape those processes and transform into stable mutations. Of these latter, only a few will target cancer genes and thus confer a growth advantage (driver mutations). The remaining mutations will be co-selected during the neoplastic process (passenger mutations). The number of driver mutations is very low (less than 20). However, that of passenger mutations is several orders of magnitude higher, ranging from 0.8 substitutions per megabase for hematological neoplasms such as CLL to 9 or 11 for lung cancer or melanoma respectively (23).

As a result, due to the specificity of the damage caused by such insults and the specific repair mechanisms used by the cell to correct the damage, mutagenic processes generate characteristic point mutation rate spectra, which are called mutational signatures. These signatures point to the mutagenic processes active in a tumor and reveal the high tissue specificity of these mutagenic mechanisms. For these analyses, passenger mutations are preferred as they are not subject to any selective process. In contrast, mutations in driver genes are highly biased as only those able to drive a cancer phenotype will be selected, whether it is *via* the LOF of a tumor suppressor gene or the gain of function of an oncogene. For the latter, mutations (predominantly missense variants) are restricted to a few codons in the gene targeting key functional residues. For tumor suppressor genes, mutations (predominantly nonsense or frameshift) will lead to a null phenotype or the synthesis of an inactive truncated protein. The mode of inactivation of *TP53* is unique compared to other tumor suppressor genes, with more than 80% of somatic and germline *TP53* alterations being missense mutations that lead to the synthesis of a stable mutant protein that accumulates in the nucleus of tumor cells (24). The classification of *TP53* as a tumor suppressor gene led to a general belief wherein the loss of *TP53* function is the sole mechanism associated with *TP53* mutations. In fact, this strong selection to maintain expression of mutants in tumors is known to have a vital role in transformation, including dominant activity (DN) and/or a gain of function (GOF), making *TP53* variants oncogenic. The distribution of mutations in the p53 protein is also unique among oncogenes and tumor suppressor genes as nearly all of the protein’s 393 amino acid residues have been the target of at least one mutation in human cancer. Each residue in the core domain (containing the DNA-binding region) has been found to be mutated at least five times in independent tumors, and up to 6,000 times for hotspot mutants.

Nevertheless, the distribution of these mutations, and therefore the landscape of *TP53* variants observed in a number of types of cancer, is very heterogenous. This aspect may result from the specificity of the insults that generate the mutations (5-methylcytosine deamination at CpG dinucleotide, UV, tobacco carcinogens or chemotherapy) or from the tissue-specific selection of *TP53* variants with a special growth advantage (25). At first glance, an analysis of the 3,914 cases of CLL in UMD_*TP53* shows a mutation profile similar to those of other cancers (**Figures 3C, D**), with 76% of missense mutations mostly localized in the DNA-binding domain of *TP53*.

The unusual feature of *TP53* mutation in CLL is the presence of a specific hot spot variant: a deletion of two nucleotides at codon 209 (c.626_627del) leading to premature termination (p.Arg209LysfsTer6) (**Figure 3E**). Frameshift variants are found all along the *TP53* gene in every type of cancer, but variant c.626_627del is highly predominant in CLL (15% of frameshift mutations in CLL compared to 1 to 2% in other cancer types). The sequence around codon 209 contains an inverted repeat that could explain its specific mutability. Furthermore, the observation of this variant in both untreated and treated patients indicates that it originates from an unknown endogenous mechanism. Although frameshift variants are usually not expressed due to NMD (nonsense-mediated mRNA decay) and protein instability, a specific selection for a truncated *TP53* cannot be formally excluded.

## Subclonality of *TP53* Mutations

Whole exome and whole genome sequencing have provided new insights into the heterogeneity and evolution of tumors, with, importantly, the detection of a high number of subclones in a single tumor (26, 27). This knowledge on the subclonality of *TP53* mutations is likely to have implications for biomarker discovery and/or cancer therapy, particularly in the era of targeted treatments. Furthermore, indications of a relationship between this heterogeneity and clinical outcomes are emerging.

*TP53* mutated subclones with variant allele frequencies (VAFs) lower than 10% (range 0.3% to 10%, depending on the study), undetectable by conventional Sanger sequencing, have been reported in multiple studies (28–33). Subclonal *TP53* variants and high VAF variants have the same profile, including similar hot spot variants. Longitudinal studies have shown that some of these clones can become more prevalent during the development of the tumor, regardless of whether the patient was treated or not. These small mutated subclones have been shown to be associated with unfavorable prognoses in some studies. However, this issue remains controversial, and there is currently no use of mutated subclones in the clinic. *TP53* classifications and the methods and cut-offs used to define low VAF clones must be harmonized to enable consensus.

Another characteristic is the high number of CLL patients with multiple *TP53* variants (**Figure 3F**). This feature appears specific to CLL; it has not been observed in other types of cancer (22). Bi-allelic *TP53* inactivation could explain two *TP53* variants but not a higher number of them (range 3 to 10) (34). This high intratumor heterogeneity has been detected in multiple independent studies and validated by specific analyses such as FASAY (functional analysis of separated alleles in yeast) and SMRT (single-molecule real-time sequencing) that confirm different allelic locations for these *TP53* variants. Like for minor subclones, most *TP53* variants identified in tumors bearing multiple *TP53* variants are truly pathogenic. The basis of this specific selection for multiple *TP53* variants during the course of CLL is currently unknown.

## *TP53* Mutation Heterogeneity and Pathogenicity

As early as the nineties, it was obvious that *TP53* mutant LOF was heterogenous. Variants were classified as “contact” or “structural,” depending on whether the substituted amino-acid acted directly on DNA interactions (p.Arg273His) or caused a general effect on the protein structure (p.Arg175His). Several classifications for variants based on *TP53* aspects have been suggested to stratify patients with *TP53* mutations but none have reached the clinic due to the high heterogeneity of the variants and the specificity of the variants among cancer types. A number of predictive tools have been developed, exploiting such information as sequence phylogenetic conservation, amino acid physicochemical properties, functional domains and structural attributes. Commonly used variant effect prediction methods include SIFT (35, 36), PolyPhen (37), GERP++ (38), Condel (39), CADD (40), fathmm (41), MutationTaster (42), MutationAssessor (43), GESPA (44) and, more recently, REVEL (45) and ENVISION (46). Several of these methods, such as fathmm, Condel, CADD and REVEL, integrate data from multiple tools to improve classification accuracy. Recent methods have used machine learning processes. Their training and validation were conducted using datasets of classified variants taken from either pathogenic (COSMIC, TCGA, GENIE, HGMD) or benign (dbSNP, gnomAD or Clinvar) variant databases. Nonetheless, for *TP53* and other genes, these various classifiers have heterogenous outcomes and no consensus for their use has been reached. GENIE uses SIFT and PolyPhen, whereas TCGA uses SIFT, PolyPhen and MutationAssessor, and COSMIC uses fathmm. When employing predicting methods based on phylogenetic conservation, tools based on amino acid physicochemical properties such as SIFT or PolyPhen should be used with great precaution as the relation of the deleteriousness of the protein predicted by these tools and any association with disease is far from being straightforward.

To solve this issue, the American College of Medical Genetics and Genomics (ACMG) and the Association for Molecular Pathology (AMP) have published standards and guidelines for the interpretation of sequence variants (47). These guidelines describe a proposition for classifying variants as “pathogenic,” “likely pathogenic”, “uncertain significance”, “likely benign” or “benign” according to a series of criteria with levels of evidence defined as “very strong”, “strong”, “moderate” or “supporting”. They have been widely adopted by clinical laboratories around the world. However, these recommendations were primarily designed for constitutional variants. Thus, their use for somatic variants requires some adjustment (48). The two main criteria used for the levels of evidence were based on population (BA1, BS1 and BS2) and functional (BS3) data, which are now fully available in UMD_*TP53*.

One of the main advantages for *TP53* over other tumor suppressor genes is the availability of a range of functional data for all possible missense mutations occurring in the coding region for the large isoform of the protein. Data from three independent large-scale saturation mutagenesis screening studies carried out in different settings (yeast or mammalian) and with different readouts (transcription, growth arrest or apoptosis) are currently available (17, 18, 49). A correlation analysis showed excellent agreement between LOF for the protein and the occurrence of these variants in different cancer types, making this criterion suitable for defining PS3 for ACMG classification (**Figure 3B**) (19). An analysis of the UMD_CLL database indicates that 91.9% of the *TP53* variants identified in CLL, whether clonal or subclonal, are classified as pathogenic and 6.7% as VUS (**Figure 3F**).

## A Snapshot of *TP53* Mutation Status in CLL

*TP53* status in tumors is complex as multiple mechanisms can impair *TP53* tumor suppression pathways. Furthermore, it is quite likely that cancer specificity plays an important role in this process due to the large diversity of *TP53* function and regulation among the various tissues. Although MDM2 expression is upregulated in numerous cancers, resulting in a loss of p53-dependent activities, its frequency in CLL is quite low. Other mechanisms, such as the dysregulation of the microRNA network that controls *TP53*, are also possible but their importance in CLL needs further investigation.

In contrast, CGDs have made it possible to identify the co-occurrence or mutual exclusivity of specific genetic events. In the former, alterations of certain combinations of genes tend to co-exist in a same tumor, whereas in the latter, mostly only one out of a group of genes is altered. Individual alterations targeting similar biological processes are believed to be mutually redundant, with one alteration being sufficient to deregulate the affected process. Identifying mutual exclusivity can therefore help to identify unknown functional interactions. In CLL, this type of analysis is averted by the important genetic heterogeneity of the tumors, showing multiple subclones with different genetic alterations. Because NGS gives a global picture of these events, defining whether or not they occur in the same cells is difficult. This problem will likely be resolved once sufficient single-cell genomic analyses have been performed.

**Figure 4:**
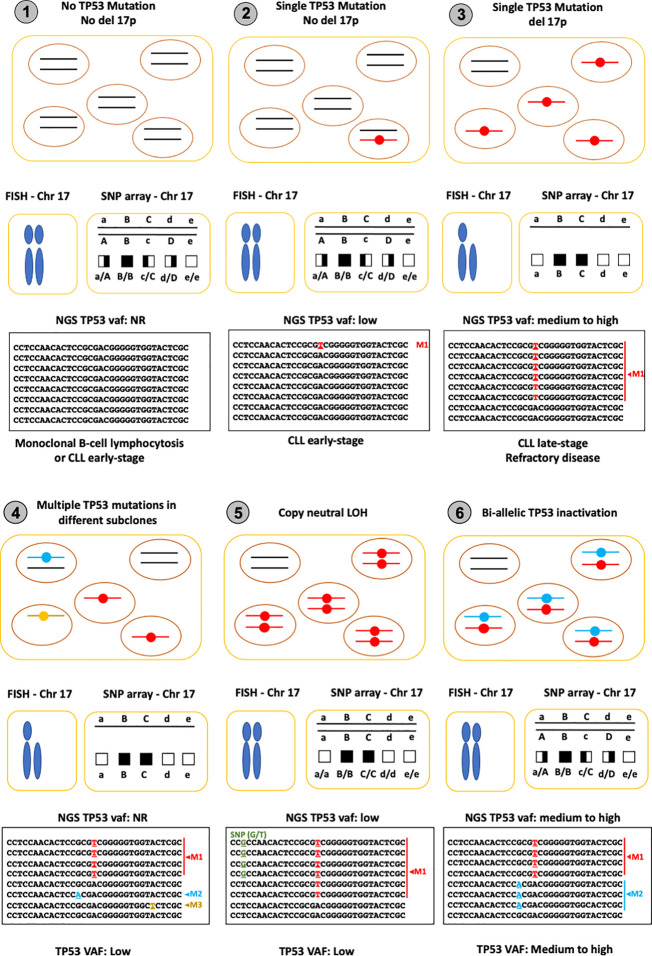
*TP53* status in CLL patients, a snapshot. The top panel displays a schematic view of the tumor with the two *TP53* alleles. The middle panel shows cytogenetic analysis performed by FISH (left) or by SNP arrays (right). The lower panel displays an example of the read alignments from NGS. 1: No *TP53* mutation: In monoclonal B-cell lymphocytosis, *TP53* mutation and 17p deletion are very rare, leading to negative results for both FISH and genetic analysis. 2: *TP53* mutation without LOH: In early stages of CLL, the frequency of *TP53* mutation is low (less than 10%) with many cases showing no LOH. Sensitive sequencing analysis with NGS is able to identify low VAF *TP53* variants (variant M1 in the lower panel). 3: *TP53* mutation with LOH: In late-stage or relapsing disease, *TP53* mutations associated with 17p deletion can be found in 30 to 50% of CLL patients. In the majority of cases, VAF is greater than 50% due to the loss of the second allele. This situation is commonly seen in CLL. 4: Multiple *TP53* and LOH: in both early and late-stage disease, FASAY (functional analysis of separated alleles of p53 on yeast) or SMRT (single-molecule, real-time sequencing) has demonstrated a high level of intratumoral heterogeneity in CLL with the presence of multiple independent subclones expressing different pathogenic *TP53* variants (M1, M2 and M3 in the lower panel). Although 17p deletion is often observed in these patients, it is difficult to determine if subclones expressing different *TP53* variants are associated with it, and even more so if the VAF of the variant is low. 5: Copy neutral LOH: Following the initial mutational inactivation of one allele, the remaining wild-type allele is deleted concurrently with the duplication of the mutated allele, leading to copy neutral LOH (cnLOH). Detecting cnLOH is difficult and thus the frequency of the event is currently unknown. Without SNP array analysis and if the VAF of the variant is lower than 50%, this situation can be misidentified as a tumor without LOH. Tumors with VAF greater than 50% without obvious 17p deletion should be checked for cnLOH. 6: Bi-allelic mutations: Inactivation of the *TP53* gene *via* different mutations in the two alleles is possible but difficult to distinguish from intratumoral heterogeneity. Although this situation is often described as plausible in many reviews, it has never been formally identified, as only single-cell sequencing would be able to validate bi-allelic *TP53* inactivation.

As shown in **Figure 4**, *TP53* status in CLL can be very heterogeneous, as the prevalence of *TP53* abnormalities, including 17p deletion and *TP53* mutations, varies across the different phases of the disease (26). Furthermore, the subclonal heterogeneity of the tumors can sometimes be misleading. Indeed, bulk NGS analyses generate an averaged picture of a given population of cells, which may result in an underestimation of their true heterogeneity. Nevertheless, a general picture emerges from the various studies. *TP53* mutations are not the prime event in CLL. In the early phase of the disease (stage 0), *TP53* mutations appear to be either absent or infrequent, but this issue needs to be carefully reevaluated using NGS assays validated for limits of detection (LOD) ranging from 0.05% to 1% (**Figure 4**, panel 1). Furthermore, because these variants are usually not associated with a deletion of the second allele, FISH or SNP arrays are not suitable for early detection analyses (**Figure 4**, panel 2). 17p deletion and complex karyotypes occur during disease progression, leading to the conventional view of CLL with a single *TP53* mutation associated with *TP53* loss of heterozygosity (LOH) (**Figure 4**, panel 3). Targeted, high-depth, NGS of *TP53* coupled with an adequate pipeline able to reach a LOD of at least 1% has led to the discovery of multiple subclones expressing different pathogenic *TP53* variants (**Figure 4**, panel 4). Why CLL has such a propensity for *TP53* mutations is currently unknown. However, it is clear that CLL depends on signals from the microenvironment and that its cells cycle between lymphoid tissue sites such as lymph nodes and peripheral blood. It is possible that the strong proliferation signals provided by the microenvironment in lymph nodes require a loss of several anti-proliferative signals such as that provided by *TP53*.

Another genetic configuration observed in CLL is copy neutral LOH (cnLOH), with the same mutation in both alleles of a given cell (**Figure 4**, panel 5). This genetic event is attributed to mitotic recombination in tumor cells where the wild-type allele is replaced by the mutant allele leading to a large region of homozygosity that can be detected early by SNP-arrays. Inversely, this situation cannot be detected by any karyotyping analyses and could be misinterpreted as heterozygous mutation if the sequencing VAF is below 50%. The situation described in panel 6 of **Figure 4** (two different mutations in the two alleles of a single cell) is theoretically possible and often described as a potential status in CLL and other tumors. However, such a situation appears to be very uncommon and has never been formally observed in CLL.

## *TP53* Clinical Considerations

*TP53* mutations have been described in CLL since the early 1990s (50, 51). An association between *TP53* mutations, drug resistance and poor clinical outcomes was first demonstrated in 1993 by El Rouby et al. and thereafter confirmed in further studies (52–54). In 2000, using FISH analysis for multiple chromosomal markers, Dohner et al. showed that 17p deletion, where the *TP53* gene is located, was an independent predictor of disease progression and survival (55). Using either 17p deletion or *TP53* mutation as a biomarker, subsequent studies confirmed this finding and resulted in *TP53*’s classification as a well-established prognostic marker furthermore able to provide pertinent information for establishing an appropriate course of treatment for patients.

The therapeutic approach for CLL carrying *TP53* mutations will be addressed in detail in another review in this series. There are, however, some biological aspects and some issues related to methodological/technical details that can be discussed here.

There is no longer a place for chemo-based regimens in patients with CLLs presenting *TP53* mutations. The introduction of novel targeted agents has greatly altered the clinical course of these patients, who now benefit from responses that were never observed during the chemo(immunotherapy) era (56). That said, even with the use of novel agents, CLL remains incurable. Patients with *TP53* disruption (*TP53* mutation/17p deletion) exhibit worse clinical outcomes compared to those without it, indicating that the management of the former is still an unmet challenge (57). This is more evident for relapsed/refractory (R/R) CLL (58–62) where data on front-line therapies are still scarce because the follow-up of clinical trials at the front line is still short (63, 64). Moreover, little is known on R/R CLL response to novel agents, a setting wherein *TP53* disruption seems to be an unfavorable prognostic/predictive factor (65, 66).

Another parameter that needs to be taken into consideration is that in both clinical trials and clinical care, *TP53* disruptions are considered equal whatever their nature. There is thus no differentiation being made between patients with monoallelic or bi-allelic aberrations, despite data suggesting that the latter may exhibit more aggressive clinical courses (33, 60). Similarly, the number or type of mutations receives no consideration as a specific clinical feature either.

Moreover, in untreated CLL with *TP53* mutations, there is a subset of patients with indolent clinical courses, which suggests that other disease- and/or patient-related parameters may alter the impact of *TP53* disruption (67, 68). Also, genomic instabilities at the chromosomal and molecular level, as well as the immunogenetic features of the clonotypic B-cell receptor, namely the somatic hypermutation status of the immunoglobulin heavy variable gene, have been proposed as factors that may aggravate or alleviate the impact of *TP53* mutations (69–72).

Finally, there is a discrepancy regarding the threshold for reporting *TP53* mutations detected by NGS in the clinical setting versus the official guidelines that merits discussion. According to the latest versions of these latter, only mutations with VAF ≥10% should be reported and used for directing treatment choice (73). This conservative approach within the official guidelines is based mainly on the fact that the clinical impact of small *TP53* clones, especially those below 5%, has not been demonstrated to date in prospective clinical trials. However, diagnostic laboratories are becoming more experienced in NGS data output management, and resultantly, clones down to 2-5% are being reported in the clinical setting and, in the majority of cases, taken into consideration for clinical decision-making.

## Remaining Challenges and Perspectives

Compared to other cancer types, the clinical value of *TP53* status in CLL has always been uncontested and it is now a required biomarker for patient stratification. It is therefore essential that *TP53* analyses be performed in a standardized manner to provide consistent data across the various clinical laboratories. For this purpose, the *TP53* Network of the European Research Initiative on Chronic Lymphocytic Leukemia (ERIC) had released a first recommendation in 2012 and updated it recently to take into account the emergence of NGS (73, 74). Nevertheless, considering the quick evolution of methodologies and the discovery of the subclonal heterogeneity of *TP53* variants with low VAF clones, a new consensus must be reached for the controversial issue of the limit of detection used to report *TP53* variants in clinical laboratories. Although conventional Sanger sequencing has been widely used in the past, it is now clear that NGS-based analysis should become mandatory for the clinical detection of low VAF clones. The current situation is unclear, with several studies suggesting that patients with low VAF *TP53* clones have the same clinical prognosis as patients with high VAF ones, and other studies unable to confirm that finding. Reaching a consensus to define a robust, clinically justified LOD will be essential for improving patient stratification. Furthermore, despite their relative infrequency, it will be important to evaluate the real incidence of multiple *TP53* subclonal mutations using adequate methodologies as well as their evolution during the course of disease and with different types of treatment. Whether or not this reservoir of heterogenous oncogenic *TP53* variants is an essential component of the plasticity of CLL remains to be addressed.

## Author Contributions

All authors listed have made a substantial, direct, and intellectual contribution to the work, and approved it for publication.

## Conflict of Interest

The authors declare that the research was conducted in the absence of any commercial or financial relationships that could be construed as a potential conflict of interest.

## Publisher’s Note

All claims expressed in this article are solely those of the authors and do not necessarily represent those of their affiliated organizations, or those of the publisher, the editors and the reviewers. Any product that may be evaluated in this article, or claim that may be made by its manufacturer, is not guaranteed or endorsed by the publisher.

## References

[1] Ingram VM. A Specific Chemical Difference Between the Globins of Normal Human and Sickle-Cell Anaemia Haemoglobin. Nature (1956) 178:792–4. doi: 10.1038/178792a0 13369537

[2] Collins FS. Positional Cloning Moves From Perditional to Traditional. Nat Genet (1995) 9:347–50. doi: 10.1038/ng0495-347 7795639

[3] Horaitis O Cotton RG. The Challenge of Documenting Mutation Across the Genome: The Human Genome Variation Society Approach. Hum Mutat (2004) 23:447–52. doi: 10.1002/humu.20038 15108276

[4] Auerbach AD Burn J Cassiman JJ Claustres M Cotton RG Cutting G. Mutation (Variation) Databases and Registries: A Rationale for Coordination of Efforts. Nat Rev Genet (2011) 12:881; discussion 881. doi: 10.1038/nrg3011-c1 22025002

[5] Dalgleish R. LSDBs and How They Have Evolved. Hum Mutat (2016) 37:532–9. doi: 10.1002/humu.22979 26919551

[6] Soussi T. Locus-Specific Databases in Cancer: What Future in a Post-Genomic Era? The TP53 LSDB Paradigm. Hum Mutat (2014) 35:643–53. doi: 10.1002/humu.22518 24478183

[7] Pinard A Miltgen M Blanchard A Mathieu H Desvignes JP Salgado D. Actionable Genes, Core Databases, and Locus-Specific Databases. Hum Mutat (2016) 37:1299–307. doi: 10.1002/humu.23112 27600092

[8] Zhang J Baran J Cros A Guberman JM Haider S Hsu J. International Cancer Genome Consortium Data Portal–A One-Stop Shop for Cancer Genomics Data. Database (Oxford) (2011) 2011:bar026. doi: 10.1093/database/bar026 21930502 PMC3263593

[9] Tate JG Bamford S Jubb HC Sondka Z Beare DM Bindal N. COSMIC: The Catalogue Of Somatic Mutations In Cancer. Nucleic Acids Res (2018) 47:D941–7. doi: 10.1093/nar/gky1015 PMC6323903 30371878

[10] Network CGAR Weinstein JN Collisson EA Mills GB Shaw KR Ozenberger BA. The Cancer Genome Atlas Pan-Cancer Analysis Project. Nat Genet (2013) 45:1113–20. doi: 10.1038/ng.2764 PMC3919969 24071849

[11] Cheok CF Verma CS Baselga J Lane DP. Translating P53 Into the Clinic. Nat Rev Clin Oncol (2011) 8:25–37. doi: 10.1038/nrclinonc.2010.174 20975744

[12] Bouaoun L Sonkin D Ardin M Hollstein M Byrnes G Zavadil J. TP53 Variations in Human Cancers: New Lessons From the IARC TP53 Database and Genomics Data. Hum Mutat (2016) 37:865–76. doi: 10.1002/humu.23035 27328919

[13] Leroy B Ballinger ML Baran-Marszak F Bond GL Braithwaite A Concin N. Recommended Guidelines for Validation, Quality Control, and Reporting of TP53 Variants in Clinical Practice. Cancer Res (2017) 77:1250–60. doi: 10.1158/0008-5472.CAN-16-2179 PMC7457206 28254861

[14] Tikkanen T Leroy B Fournier JL Risques RA Malcikova J Soussi T. Seshat: A Web Service for Accurate Annotation, Validation, and Analysis of TP53 Variants Generated by Conventional and Next-Generation Sequencing. Hum Mutat (2018) 39:925–33. doi: 10.1002/humu.23543 29696732

[15] Soussi T Kato S Levy PP Ishioka C. Reassessment of the TP53 Mutation Database in Human Disease by Data Mining With a Library of TP53 Missense Mutations. Hum Mutat (2005) 25:6–17. doi: 10.1002/humu.20114 15580553

[16] Soussi T Asselain B Hamroun D Kato S Ishioka C Claustres M. Meta-Analysis of the P53 Mutation Database for Mutant P53 Biological Activity Reveals a Methodologic Bias in Mutation Detection. Clin Cancer Res (2006) 12:62–9. doi: 10.1158/1078-0432.CCR-05-0413 16397025

[17] Kotler E Shani O Goldfeld G Lotan-Pompan M Tarcic O Gershoni A. A Systematic P53 Mutation Library Links Differential Functional Impact to Cancer Mutation Pattern and Evolutionary Conservation. Mol Cell (2018) 71:178–190.e8. doi: 10.1016/j.molcel.2018.06.012 29979965

[18] Giacomelli AO Yang X Lintner RE McFarland JM Duby M Kim J. Mutational Processes Shape the Landscape of TP53 Mutations in Human Cancer. Nat Genet (2018) 50:1381–7. doi: 10.1038/s41588-018-0204-y PMC6168352 30224644

[19] Carbonnier V Leroy B Rosenberg S Soussi T. Comprehensive Assessment of TP53 Loss of Function Using Multiple Combinatorial Mutagenesis Libraries. Sci Rep (2020) 10:20368. doi: 10.1038/s41598-020-74892-2 33230179 PMC7683535

[20] Li X Dumont P Della Pietra A Shetler C Murphy ME. The Codon 47 Polymorphism in P53 Is Functionally Significant. J Biol Chem (2005) 280:24245–51. doi: 10.1074/jbc.M414637200 15851479

[21] Doffe F Carbonnier V Tissier M Leroy B Martins I Mattsson JSM. Identification and Functional Characterization of New Missense SNPs in the Coding Region of the TP53 Gene. Cell Death Differ (2021) 28:1477–92. doi: 10.1038/s41418-020-00672-0 PMC8166836 33257846

[22] Donehower LA Soussi T Korkut A Liu Y Schultz A Cardenas M. Integrated Analysis of TP53 Gene and Pathway Alterations in The Cancer Genome Atlas. Cell Rep (2019) 28:1370–84.e5. doi: 10.1016/j.celrep.2019.07.001 PMC7546539 31365877

[23] Ding L Bailey MH Porta-Pardo E Thorsson V Colaprico A Bertrand D. Perspective on Oncogenic Processes at the End of the Beginning of Cancer Genomics. Cell (2018) 173:305–320.e10. doi: 10.1016/j.cell.2018.03.033 29625049 PMC5916814

[24] Soussi T Wiman KG. TP53: An Oncogene in Disguise. Cell Death Differ (2015) 22:1239–49. doi: 10.1038/cdd.2015.53 PMC4495363 26024390

[25] Leroy B Anderson M Soussi T. TP53 Mutations in Human Cancer: Database Reassessment and Prospects for the Next Decade. Hum Mutat (2014) 35:672–88. doi: 10.1002/humu.22552 24665023

[26] Lazarian G Guièze R Wu CJ. Clinical Implications of Novel Genomic Discoveries in Chronic Lymphocytic Leukemia. J Clin Oncol (2017) 35:984–93. doi: 10.1200/JCO.2016.71.0822 PMC5559883 28297623

[27] Fabbri G Dalla-Favera R. The Molecular Pathogenesis of Chronic Lymphocytic Leukaemia. Nat Rev Cancer (2016) 16:145–62. doi: 10.1038/nrc.2016.8 26911189

[28] Rossi D Khiabanian H Spina V Ciardullo C Bruscaggin A Famà R. Clinical Impact of Small TP53 Mutated Subclones in Chronic Lymphocytic Leukemia. Blood (2014) 123:2139–47. doi: 10.1182/blood-2013-11-539726 PMC4017291 24501221

[29] Bomben R Rossi FM Vit F Bittolo T D’Agaro T Zucchetto A. TP53 Mutations With Low Variant Allele Frequency Predict Short Survival in Chronic Lymphocytic Leukemia. Clin Cancer Res (2021) 27:5566–75. doi: 10.1158/1078-0432.CCR-21-0701 34285062

[30] Nadeu F Delgado J Royo C Baumann T Stankovic T Pinyol M. Clinical Impact of Clonal and Subclonal TP53, SF3B1, BIRC3, NOTCH1, and ATM Mutations in Chronic Lymphocytic Leukemia. Blood (2016) 127:2122–30. doi: 10.1182/blood-2015-07-659144 PMC4912011 26837699

[31] Brieghel C Kinalis S Yde CW Schmidt AY Jønson L Andersen MA. Deep Targeted Sequencing of TP53 in Chronic Lymphocytic Leukemia: Clinical Impact at Diagnosis and at Time of Treatment. Haematologica (2019) 104:789–96. doi: 10.3324/haematol.2018.195818 PMC6442964 30514802

[32] Stengel A Kern W Haferlach T Meggendorfer M Fasan A Haferlach C. The Impact of TP53 Mutations and TP53 Deletions on Survival Varies Between AML, ALL, MDS and CLL: An Analysis of 3307 Cases. Leukemia (2017) 31:705–11. doi: 10.1038/leu.2016.263 27680515

[33] Blakemore SJ Clifford R Parker H Antoniou P Stec-Dziedzic E Larrayoz M. Clinical Significance of TP53, BIRC3, ATM and MAPK-ERK Genes in Chronic Lymphocytic Leukaemia: Data From the Randomised UK LRF CLL4 Trial. Leukemia (2020) 34:1760–74. doi: 10.1038/s41375-020-0723-2 PMC7326706 32015491

[34] Malcikova J Stano-Kozubik K Tichy B Kantorova B Pavlova S Tom N. Detailed Analysis of Therapy-Driven Clonal Evolution of TP53 Mutations in Chronic Lymphocytic Leukemia. Leukemia (2015) 29:877–85. doi: 10.1038/leu.2014.297 PMC4396398 25287991

[35] Kumar P Henikoff S Ng PC. Predicting the Effects of Coding non-Synonymous Variants on Protein Function Using the SIFT Algorithm. Nat Protoc (2009) 4:1073–81. doi: 10.1038/nprot.2009.86 19561590

[36] Ng PC Henikoff S. Predicting Deleterious Amino Acid Substitutions. Genome Res (2001) 11:863–74. doi: 10.1101/gr.176601 PMC311071 11337480

[37] Adzhubei IA Schmidt S Peshkin L Ramensky VE Gerasimova A Bork P. A Method and Server for Predicting Damaging Missense Mutations. Nat Methods (2010) 7:248–9. doi: 10.1038/nmeth0410-248 PMC2855889 20354512

[38] Davydov EV Goode DL Sirota M Cooper GM Sidow A Batzoglou S. Identifying a High Fraction of the Human Genome to be Under Selective Constraint Using GERP++. PloS Comput Biol (2010) 6:e1001025. doi: 10.1371/journal.pcbi.1001025 21152010 PMC2996323

[39] González-Pérez A López-Bigas N. Improving the Assessment of the Outcome of Nonsynonymous SNVs With a Consensus Deleteriousness Score, Condel. Am J Hum Genet (2011) 88:440–9. doi: 10.1016/j.ajhg.2011.03.004 PMC3071923 21457909

[40] Kircher M Witten DM Jain P O’Roak BJ Cooper GM Shendure J. A General Framework for Estimating the Relative Pathogenicity of Human Genetic Variants. Nat Genet (2014) 46:310–5. doi: 10.1038/ng.2892 PMC3992975 24487276

[41] Shihab HA Rogers MF Gough J Mort M Cooper DN Day IN. An Integrative Approach to Predicting the Functional Effects of Non-Coding and Coding Sequence Variation. Bioinformatics (2015) 31:1536–43. doi: 10.1093/bioinformatics/btv009 PMC4426838 25583119

[42] Schwarz JM Rödelsperger C Schuelke M Seelow D. MutationTaster Evaluates Disease-Causing Potential of Sequence Alterations. Nat Methods (2010) 7:575–6. doi: 10.1038/nmeth0810-575 20676075

[43] Reva B Antipin Y Sander C. Predicting the Functional Impact of Protein Mutations: Application to Cancer Genomics. Nucleic Acids Res (2011) 39:e118. doi: 10.1093/nar/gkr407 21727090 PMC3177186

[44] Khurana JK Reeder JE Shrimpton AE Thakar J. GESPA: Classifying nsSNPs to Predict Disease Association. BMC Bioinf (2015) 16:228. doi: 10.1186/s12859-015-0673-2 PMC4513380 26206375

[45] Ioannidis NM Rothstein JH Pejaver V Middha S McDonnell SK Baheti S. REVEL: An Ensemble Method for Predicting the Pathogenicity of Rare Missense Variants. Am J Hum Genet (2016) 99:877–85. doi: 10.1016/j.ajhg.2016.08.016 PMC5065685 27666373

[46] Gray VE Hause RJ Luebeck J Shendure J Fowler DM. Quantitative Missense Variant Effect Prediction Using Large-Scale Mutagenesis Data. Cell Syst (2018) 6:116–124.e3. doi: 10.1016/j.cels.2017.11.003 29226803 PMC5799033

[47] Richards S Aziz N Bale S Bick D Das S Gastier-Foster J. Standards and Guidelines for the Interpretation of Sequence Variants: A Joint Consensus Recommendation of the American College of Medical Genetics and Genomics and the Association for Molecular Pathology. Genet Med (2015) 17:405–24. doi: 10.1038/gim.2015.30 PMC4544753 25741868

[48] Li MM Datto M Duncavage EJ Kulkarni S Lindeman NI Roy S. Standards and Guidelines for the Interpretation and Reporting of Sequence Variants in Cancer: A Joint Consensus Recommendation of the Association for Molecular Pathology, American Society of Clinical Oncology, and College of American Pathologists. J Mol Diagn (2017) 19:4–23. doi: 10.1016/j.jmoldx.2016.10.002 27993330 PMC5707196

[49] Kato S Han SY Liu W Otsuka K Shibata H Kanamaru R. Understanding the Function-Structure and Function-Mutation Relationships of P53 Tumor Suppressor Protein by High-Resolution Missense Mutation Analysis. Proc Natl Acad Sci USA (2003) 100:8424–9. doi: 10.1073/pnas.1431692100 PMC166245 12826609

[50] Gaidano G Ballerini P Gong JZ Inghirami G Neri A Newcomb EW. P53 Mutations in Human Lymphoid Malignancies: Association With Burkitt Lymphoma and Chronic Lymphocytic Leukemia. Proc Natl Acad Sci USA (1991) 88:5413–7. doi: 10.1073/pnas.88.12.5413 PMC51883 2052620

[51] Fenaux P Preudhomme C Lai JL Quiquandon I Jonveaux P Vanrumbeke M. Mutations of the P53 Gene in B-Cell Chronic Lymphocytic Leukemia: A Report on 39 Cases With Cytogenetic Analysis. Leukemia (1992) 6:246–50. 1588788

[52] el Rouby S Thomas A Costin D Rosenberg CR Potmesil M Silber R. P53 Gene Mutation in B-Cell Chronic Lymphocytic Leukemia Is Associated With Drug Resistance and is Independent of MDR1/MDR3 Gene Expression. Blood (1993) 82:3452–9. doi: 10.1182/blood.V82.11.3452.3452 8241511

[53] Dohner H Fischer K Bentz M Hansen K Benner A Cabot G. P53 Gene Deletion Predicts for Poor Survival and Non-Response to Therapy With Purine Analogs in Chronic B-Cell Leukemias. Blood (1995) 85:1580–9. doi: 10.1182/blood.V85.6.1580.bloodjournal8561580 7888675

[54] Wattel E Preudhomme C Hecquet B Vanrumbeke M Quesnel B Dervite I. P53 Mutations Are Associated With Resistance to Chemotherapy and Short Survival in Hematologic Malignancies. Blood (1994) 84:3148–57. doi: 10.1182/blood.V84.9.3148.3148 7949187

[55] Döhner H Stilgenbauer S Benner A Leupolt E Kröber A Bullinger L. Genomic Aberrations and Survival in Chronic Lymphocytic Leukemia. N Engl J Med (2000) 343:1910–6. doi: 10.1056/NEJM200012283432602 11136261

[56] Burger JA. Treatment of Chronic Lymphocytic Leukemia. N Engl J Med (2020) 383:460–73. doi: 10.1056/NEJMra1908213 32726532

[57] Ding W. The Ongoing Unmet Needs in Chronic Lymphocytic Leukemia: TP53 Disruption, Richter, and Beyond. Hematol Oncol Clin North Am (2021) 35:739–59. doi: 10.1016/j.hoc.2021.04.001 34174984

[58] Stilgenbauer S Eichhorst B Schetelig J Hillmen P Seymour JF Coutre S. Venetoclax for Patients With Chronic Lymphocytic Leukemia With 17p Deletion: Results From the Full Population of a Phase II Pivotal Trial. J Clin Oncol (2018) 36:1973–80. doi: 10.1200/JCO.2017.76.6840 29715056

[59] Stilgenbauer S Eichhorst B Schetelig J Coutre S Seymour JF Munir T. Venetoclax in Relapsed or Refractory Chronic Lymphocytic Leukaemia With 17p Deletion: A Multicentre, Open-Label, Phase 2 Study. Lancet Oncol (2016) 17:768–78. doi: 10.1016/S1470-2045(16)30019-5 27178240

[60] Brown JR Hillmen P O’Brien S Barrientos JC Reddy NM Coutre SE. Extended Follow-Up and Impact of High-Risk Prognostic Factors From the Phase 3 RESONATE Study in Patients With Previously Treated CLL/SLL. Leukemia (2018) 32:83–91. doi: 10.1038/leu.2017.175 28592889 PMC5770586

[61] Byrd JC Furman RR Coutre SE Flinn IW Burger JA Blum KA. Targeting BTK With Ibrutinib in Relapsed Chronic Lymphocytic Leukemia. N Engl J Med (2013) 369:32–42. doi: 10.1056/NEJMoa1215637 23782158 PMC3772525

[62] Byrd JC Furman RR Coutre SE Flinn IW Burger JA Blum K. Ibrutinib Treatment for First-Line and Relapsed/Refractory Chronic Lymphocytic Leukemia: Final Analysis of the Pivotal Phase Ib/II PCYC-1102 Study. Clin Cancer Res (2020) 26:3918–27. doi: 10.1158/1078-0432.CCR-19-2856 PMC8175012 32209572

[63] Fischer K Al-Sawaf O Bahlo J Fink AM Tandon M Dixon M. Venetoclax and Obinutuzumab in Patients With CLL and Coexisting Conditions. N Engl J Med (2019) 380:2225–36. doi: 10.1056/NEJMoa1815281 31166681

[64] Burger JA Sivina M Jain N Kim E Kadia T Estrov Z. Randomized Trial of Ibrutinib vs Ibrutinib Plus Rituximab in Patients With Chronic Lymphocytic Leukemia. Blood (2019) 133:1011–9. doi: 10.1182/blood-2018-10-879429 PMC6405333 30530801

[65] Roberts AW Davids MS Pagel JM Kahl BS Puvvada SD Gerecitano JF. Targeting BCL2 With Venetoclax in Relapsed Chronic Lymphocytic Leukemia. N Engl J Med (2016) 374:311–22. doi: 10.1056/NEJMoa1513257 PMC7107002 26639348

[66] Roberts AW Ma S Kipps TJ Coutre SE Davids MS Eichhorst B. Efficacy of Venetoclax in Relapsed Chronic Lymphocytic Leukemia Is Influenced by Disease and Response Variables. Blood (2019) 134:111–22. doi: 10.1182/blood.2018882555 PMC6624969 31023700

[67] Baliakas P Jeromin S Iskas M Puiggros A Plevova K Nguyen-Khac F. Cytogenetic Complexity in Chronic Lymphocytic Leukemia: Definitions, Associations, and Clinical Impact. Blood (2019) 133:1205–16. doi: 10.1182/blood-2018-09-873083 PMC6509568 30602617

[68] Leeksma AC Baliakas P Moysiadis T Puiggros A Plevova K Van Der Kevie-Kersemaekers AM. Genomic Arrays Identify High-Risk Chronic Lymphocytic Leukemia With Genomic Complexity: A Multi-Center Study. Haematologica (2021) 106:87–97. doi: 10.3324/haematol.2019.239947 31974198 PMC7776256

[69] Baliakas P Moysiadis T Hadzidimitriou A Xochelli A Jeromin S Agathangelidis A. Tailored Approaches Grounded on Immunogenetic Features for Refined Prognostication in Chronic Lymphocytic Leukemia. Haematologica (2019) 104:360–9. doi: 10.3324/haematol.2018.195032 PMC6355487 30262567

[70] Sutton LA Hadzidimitriou A Baliakas P Agathangelidis A Langerak AW Stilgenbauer S. Immunoglobulin Genes in Chronic Lymphocytic Leukemia: Key to Understanding the Disease and Improving Risk Stratification. Haematologica (2017) 102:968–71. doi: 10.3324/haematol.2017.165605 PMC5451327 28566340

[71] Ljungström V Cortese D Young E Pandzic T Mansouri L Plevova K. Whole-Exome Sequencing in Relapsing Chronic Lymphocytic Leukemia: Clinical Impact of Recurrent RPS15 Mutations. Blood (2016) 127:1007–16. doi: 10.1182/blood-2015-10-674572 PMC4768426 26675346

[72] Yu L Kim HT Kasar S Benien P Du W Hoang K. Survival of Del17p CLL Depends on Genomic Complexity and Somatic Mutation. Clin Cancer Res (2017) 23:735–45. doi: 10.1158/1078-0432.CCR-16-0594 PMC5467311 27503198

[73] Malcikova J Tausch E Rossi D Sutton LA Soussi T Zenz T. ERIC Recommendations for TP53 Mutation Analysis in Chronic Lymphocytic Leukemia-Update on Methodological Approaches and Results Interpretation. Leukemia (2018) 32:1070–80. doi: 10.1038/s41375-017-0007-7 PMC5940638 29467486

[74] Pospisilova S Gonzalez D Malcikova J Trbusek M Rossi D Kater AP. ERIC Recommendations on TP53 Mutation Analysis in Chronic Lymphocytic Leukemia. Leukemia (2012) 26:1458–61. doi: 10.1038/leu.2012.25 22297721

